# Lipid metabolites and nitric oxide production in the cerebrospinal fluid and plasma of dogs with meningoencephalitis of unknown origin and idiopathic epilepsy: a pilot study

**DOI:** 10.3389/fvets.2024.1397868

**Published:** 2024-06-25

**Authors:** Tomohiro Yonezawa, Shinya Takenouchi, Tomoki Motegi, Michiyo Miyazaki, Nanae Nagata, Koji Kobayashi, Masaki Yamada, Takahisa Murata

**Affiliations:** ^1^Laboratory of Veterinary Clinical Pathobiology, Graduate School of Agricultural and Life Sciences, The University of Tokyo, Tokyo, Japan; ^2^Veterinary Medical Center of the University of Tokyo, Graduate School of Agricultural and Life Sciences, The University of Tokyo, Tokyo, Japan; ^3^Laboratory of Animal Radiology, Graduate School of Agricultural and Life Sciences, The University of Tokyo, Tokyo, Japan; ^4^Laboratory of Food and Animal Systemics, Graduate School of Agricultural and Life Sciences, The University of Tokyo, Tokyo, Japan; ^5^Solutions COE, Shimadzu Corporation, Kyoto, Japan; ^6^Laboratory of Veterinary Pharmacology, Graduate School of Agricultural and Life Sciences, The University of Tokyo, Tokyo, Japan

**Keywords:** epileptic seizures, nitric oxide, prostaglandin, arachidonic acid, cerebrospinal fluid

## Abstract

**Introduction:**

Idiopathic epilepsy (IE) and meningoencephalomyelitis of unknown origin (MUO) are common causes of brain diseases leading to seizures in dogs. In this study, the concentrations of 196 lipid metabolites and nitrogen oxide (NO) production in the cerebrospinal fluid (CSF) and plasma of dogs with MUO or IE were measured using a LC-MS/MS and a NOx analyzer, respectively.

**Methods:**

Nine clinically healthy dogs and 11 and 12 dogs with IE and MUO, respectively, were included in the study.

**Results:**

Lipid analysis revealed variations in the levels of four and six lipid metabolites in CSF and plasma, respectively, between the groups. The levels of 6-keto-prostaglandin (PG) F1_α_ (PGF1_α_), 20-carboxy arachidonic acid (20-carboxy-AA), 9-hydroxyoctadecadienoic acid, and lyso-platelet-activating factor were high in the CSF of dogs with MUO. In addition, the plasma levels of 11,12-dihydroxyeicosatrienoic acid, 20-carboxy-AA, and oleoylethanolamide were high in dogs with IE, and those of PGF1_α_ were high in dogs with MUO. NO production levels were high in CSF but not in plasma in dogs with MUO or IE.

**Discussion:**

It remains unknown whether these changes represent the cause or effect of diseases of the central nervous system; however, lipid metabolites and NO production in CSF and plasma may be used as diagnostic biomarkers and could be exploited for treating idiopathic or inflammatory epilepsy in dogs.

## 1 Introduction

Epilepsy is a common chronic and functional brain disorder in mammals, including humans and dogs, characterized by recurrent seizures. In dogs, epilepsy has been estimated to occur in 0.5%−5.7% of the general population (1–3). Meningoencephalomyelitis of unknown origin (MUO) and idiopathic epilepsy (IE) are common causes of brain diseases with seizures in dogs. MUO is a well-known inflammatory disease of the brain (4). Although a few cases of MUO are caused by autoimmune inflammation (5–7), the pathophysiology of most cases remains unknown and encompasses a group of idiopathic diseases (4). Although necropsy and histopathological approaches are required for a definitive diagnosis, clinicians tend to make presumptive diagnoses based on clinical diagnostic criteria (4).

In contrast, IE is defined as recurrent unprovoked seizures with no identifiable underlying cause (8). IE is a major type of canine epilepsy reported in 25%−48% of epileptic dogs (1). Moreover, it is considered an exclusion diagnosis based on recurrent epileptic seizures with no evidence of structural epilepsy or reactive seizures on magnetic resonance imaging (MRI) or cerebrospinal fluid (CSF) analysis (8). These criteria make it difficult to diagnose IE definitively. Moreover, performing an MRI test on dogs requires moving the animal to a reference hospital with an MRI apparatus, incurring considerable medical expenses and requiring systemic anesthesia or sedation. Therefore, research to identify new diagnostic biomarkers for confirming MUO and IE is ongoing in small animal veterinary practice.

Lipid mediators are small molecules derived from polyunsaturated fatty acids via enzymatic or nonenzymatic oxidation processes and regulate the progression and resolution of inflammation. In a few diseases of the central nervous system (CNS), the levels of major inflammatory lipid mediators, i.e., prostaglandins (PGs), and other bioactive lipid mediators change to regulate protein–lipid interactions and transmembrane and trans-synaptic signaling (9). Several lipid mediators, such as platelet-activating factors (PAFs) induced by oxygen stress, are implicated in neuronal cellular injury and neurodegeneration (10, 11). In humans, nitric oxide (NO) changes local cerebral blood flow during seizures (12). Although several reports have suggested a relationship between individual lipid substances and CNS diseases, there are no reports on the comprehensive analyses of lipid metabolites in the CSF and plasma of dogs and humans with epileptic seizures. Spontaneous canine epilepsy has been discussed as a translational model of human epilepsy (3, 13, 14). The accumulation of pathophysiological data from studies on dogs with epilepsy could lead to the development of new and precise diagnostic criteria and help elucidate the fundamental background of epilepsy in both dogs and humans.

In this study, we analyzed the variations in the concentrations of various lipid metabolites and NO production in the CSF and plasma of dogs with seizure disorders, caused by MUO or IE. The pathophysiological significance and clinical value of lipid measurements in the CSF are discussed by comparing the profile, clinical history, treatment, MR images, and routine CSF tests in dogs with MUO or IE with those in healthy dogs.

## 2 Methods

### 2.1 Animals and sample collection

The clinical samples were collected retrospectively at the Veterinary Medical Center, University of Tokyo, covering the period between October 2018 and April 2020. All patients were client-owned dogs. The CSF and plasma samples from the clinical cases were initially collected for diagnosis by veterinarians. When the residual volumes of the samples were substantial after initial use, they were stored and approved for use in research. Written informed consent was obtained from all owners for the use of dogs for research post-clinical use, and ethical approval was obtained from the Veterinary Ethical Review Committee of the University of Tokyo (P18-115). The clinically healthy dogs in the control group were maintained at the Laboratory of Veterinary Surgery, University of Tokyo. The samples from those dogs were collected by trained veterinarians under the appropriate anesthesia. The experiment for CSF collection from those dogs was approved by the Animal Care and Use Committee of the Graduate School of Agricultural and Life Sciences, University of Tokyo (P18-115).

All patients were clinically diagnosed with IE and MUO at our hospital. The procedure used for diagnosing IE at the Tier II confidence level was based on the International Veterinary Epilepsy Task Force Consensus Proposal 2015 (8). No abnormalities were evident in neurological tests, cranial MRI, or CSF examination, and reactive seizures were ruled out based on clinical history, physical examination, and blood examinations. Moreover, MUO was diagnosed based on a review by Granger et al. (15) and Flegel (16). CSF collection was attempted in all the cases without those who had high risks or did not get the owner's approval. It was determined as a high-risk case when the case had signs related to brain edema, increased cerebral pressure, or orthopedic abnormalities near the occipital skull. The samples were also excluded when the CSF was contaminated with blood, or the amount was not substantial. For CSF examination, the specific gravity, cell number, and microscopic observation of the sediment were performed. For blood examination, complete blood count, total protein, albumin, blood urea nitrogen, creatinine, alanine aminotransferase, alkaline phosphatase, ammonia, glucose, calcium, phosphate, and electrolytes were measured.

Plasma and CSF samples were obtained during clinically indicated procedures. Intravenously collected blood samples were placed in heparinized tubes and centrifuged at 500 × *g* for 5 min. The supernatants were stored at −80°C as plasma samples. The CSF samples were collected under isoflurane anesthesia using the cerebellomedullary cisternal puncture method with a 23- or 25-gauge needle. Following routine analyses, supernatants were collected after centrifugation and stored at −80°C as the CSF samples.

### 2.2 Comprehensive lipid analysis using LC-MS/MS

After the CSF samples were centrifuged at 20,000 × *g* for 10 min at 4°C, 50 μl of supernatant was mixed with 750 μl of 1% formic acid water and 10 μl of internal standard solution. The mixed solutions were applied to a solid-phase extraction cartridge, OASIS HLB μElution plate (Waters, Milford, MA, USA), preconditioned with 200 μl of methanol and distilled water. Plasma samples were centrifuged in the same way as that described for CSF samples, and 100 μl of supernatant was deproteinized by mixing with 100 μl of organic solvent (methanol: acetonitrile = 1:1, v/v, containing 5N HCl) and 10 μl of internal standard solution. After centrifugation, 100 μl of supernatant was mixed with 700 μl of 1% formic acid water. Mixed solutions were loaded onto Oasis PRiME HLB plate (Waters, USA).

After washing with 200 μl of distilled water and 200 μl of hexane, the lipid fractions from CSF or plasma samples were eluted with 50 μl of methanol. The eluted sample solutions were filtered through an Ultrafree-MC Centrifugal Filter (Merck KGaA, Darmstadt, Germany). The collected analytes were used for measurements.

The 5-μl analytes were injected into a high-performance liquid chromatograph (Nexera X2; Shimadzu Corporation, Kyoto, Japan) fitted with a mass spectrometer (LCMS-8060; Shimadzu Corporation, Japan). We measured 196 types of lipid mediators and analyzed them using the Method Package for Lipid Mediators version 3 with LabSolutions software (Shimadzu Corporation, Japan). Each lipid was identified based on its retention time and selected reaction-monitoring ion transitions. The levels of each lipid mediator were assessed by comparing the peak area ratio calculated by dividing the peak area of each metabolite with that of the internal standard.

### 2.3 Measurement of NO production levels

Approximately 30 μl of CSF or plasma samples were used for measurement of nitrite (NO${\;}_{2}^{-}$) and nitrate (NO${\;}_{3}^{-}$) ions, stable metabolites of NO, using ENO-20 NOx analyzer (Eicom, Kyoto, Japan) following the manufacturer's instructions. The standard solutions used were NaNO_2_ and NaNO_3_ (Wako, Osaka, Japan).

### 2.4 Statistical analyses

All statistical analyses were conducted using XLSTAT Life Science (version 2021.2.2.1141; AddinSoft, Paris, France) as an add-in to MS Excel (Microsoft Corporation, Redmond, WA, USA). The Kruskal–Wallis's test, Steel–Dwass multiple comparison test, and Pearson's correlation analysis were used to compare the groups. For subgroup analysis to assess the confounding factors, the Mann-Whitney analysis was used. The significance level was set at *p*-value < 0.05 for all statistical comparisons.

## 3 Results

### 3.1 Sample information

In total, 32 dogs, including 11 with IE, 12 with MUO, and nine clinically healthy dogs, were used in this study (Supplementary Table 1). The breeds included beagles (*n* = 9), chihuahuas (*n* = 9), toy poodles (*n* = 6), maltese poodles (*n* = 2), papillons (*n* = 2), pugs (*n* = 2), yorkshire terriers (*n* = 1), and mixed breeds (*n* = 1). Moreover, there were 11 males (seven neutered) and 21 females (14 neutered). Their median age was 5.0 years, with a range of 1–14 years, and their median body weight was 4.2 kg, with a range of 1.9 to 11.3 kg. There are differences in breeds and gender between groups, and their ages and body weights were also different between the groups as shown in Supplementary Table 2. Of the 23 symptomatic dogs, 13 had generalized seizures, three had focal seizures, and seven did not have seizures but had other neurological abnormalities, such as paresis, lameness, head tilt, and blindness. The median period post-onset of the last seizure was 12.5 days, ranging from 1 to 26 days. Seven of 11 dogs diagnosed with IE and four of 12 dogs diagnosed with MUO were prescribed antiepileptic drugs (AED) in the past. Prednisolone had also been prescribed to one dog with IE and seven dogs with MUO. Traumatic samples were not specifically excluded because no traumatic signs were observed in all the CSF samples in this study. A part of the CSF sample was quickly used for cell counting. Cell numbers in CSF ranged from 0 to 6 cells/μl (median; 2.0 cells/μl) for IE, and 0–112 cells/μl (median; 13.5 cells/μl) for MUO. The obtained CSF was processed based on the guidelines of the International Veterinary Epilepsy Task Force (8).

### 3.2 Profiles of lipid production in CSF

LC-MS/MS was administered according to the same method described in Takenouchi et al. (17). In this study, 196 categories of lipid metabolites in the CSF and plasma were measured using LC-MS/MS. A few samples were not collected as shown in Supplementary Table 1. A substantial amount of plasma from 13 dogs and CSF samples from four dogs were not stored. Of these 196 lipids, 89 were not detected in the CSF, whereas 69 were within the reference range.

**Figure 1:**
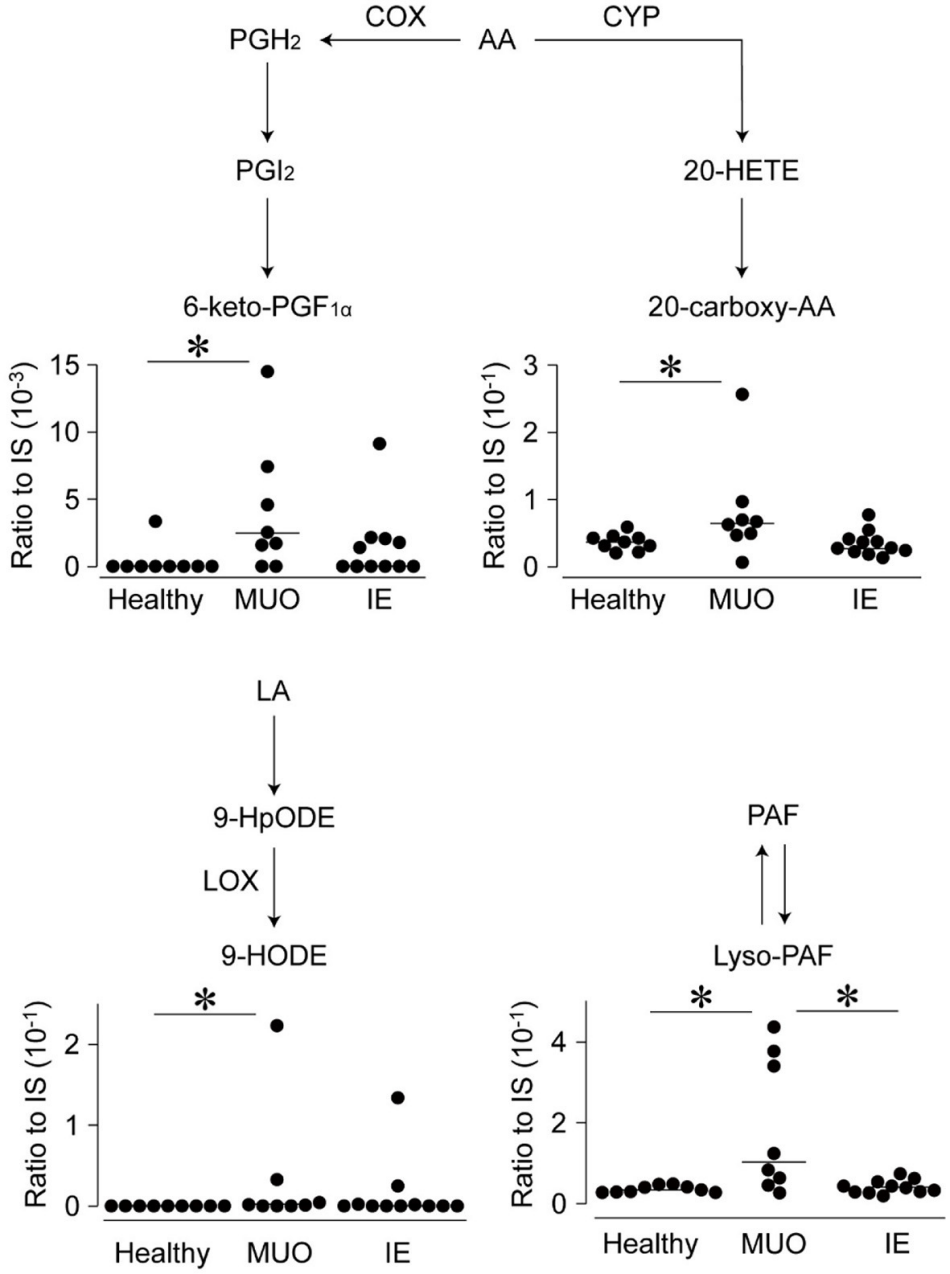
Levels of lipid metabolites in the cerebrospinal fluid (CSF) of dogs with diseases of central nervous system (CNS) on the brief lipid metabolic map. The levels of lipid metabolites, which were significantly different between the groups, are shown on the brief map. Dots represent individual values, and horizontal bars represent the medians of each group. All values are represented as ratios to internal standards (IS). Abbreviations on the side of arrows indicate the mediators of those pathways. AA, arachidonic acid; COX, cyclooxygenase; CYP, cytochrome P450; HETE, hydroxyeicosatetraenoic acid; HODE, hydroxy-10E,12Z-octadecadienoic acid; HpODE, hydroperoxy-10E,12Z-octadecadienoic acid; IE, idiopathic epilepsy; LA, linoleic acid; LOX, lipoxygenase; MUO, meningoencephalomyelitis of unknown origin; PAF, platelet-activating factor; PGF1_α_, prostaglandin F1 alpha. *indicates statistical significance at *p* < 0.05.

Among these, the levels of four types of lipids in the CSF differed significantly between the groups. Figure 1 shows the levels of these lipid metabolites, along with a schematic diagram of lipid metabolism. The levels of 6-keto-PGF_1α_, a metabolite of PGI_2_, in CSF, were higher in dogs with MUO than in healthy dogs. Similarly, the levels of 20-carboxy arachidonic acid (20-carboxy-AA), 9-hydroxyoctadecadienoic acid (9-HODE), and lyso-PAF were significantly higher in the MUO group than in the control group. The levels of lyso-PAF were also significantly higher in dogs with MUO than in dogs with IE (*p* < 0.05) and correlated positively with the cell number in CSF of MUO cases (*R* = 0.926, *p* < 0.001; Supplementary Figure 1).

### 3.3 Profiles of lipid production in plasma

**Figure 2:**
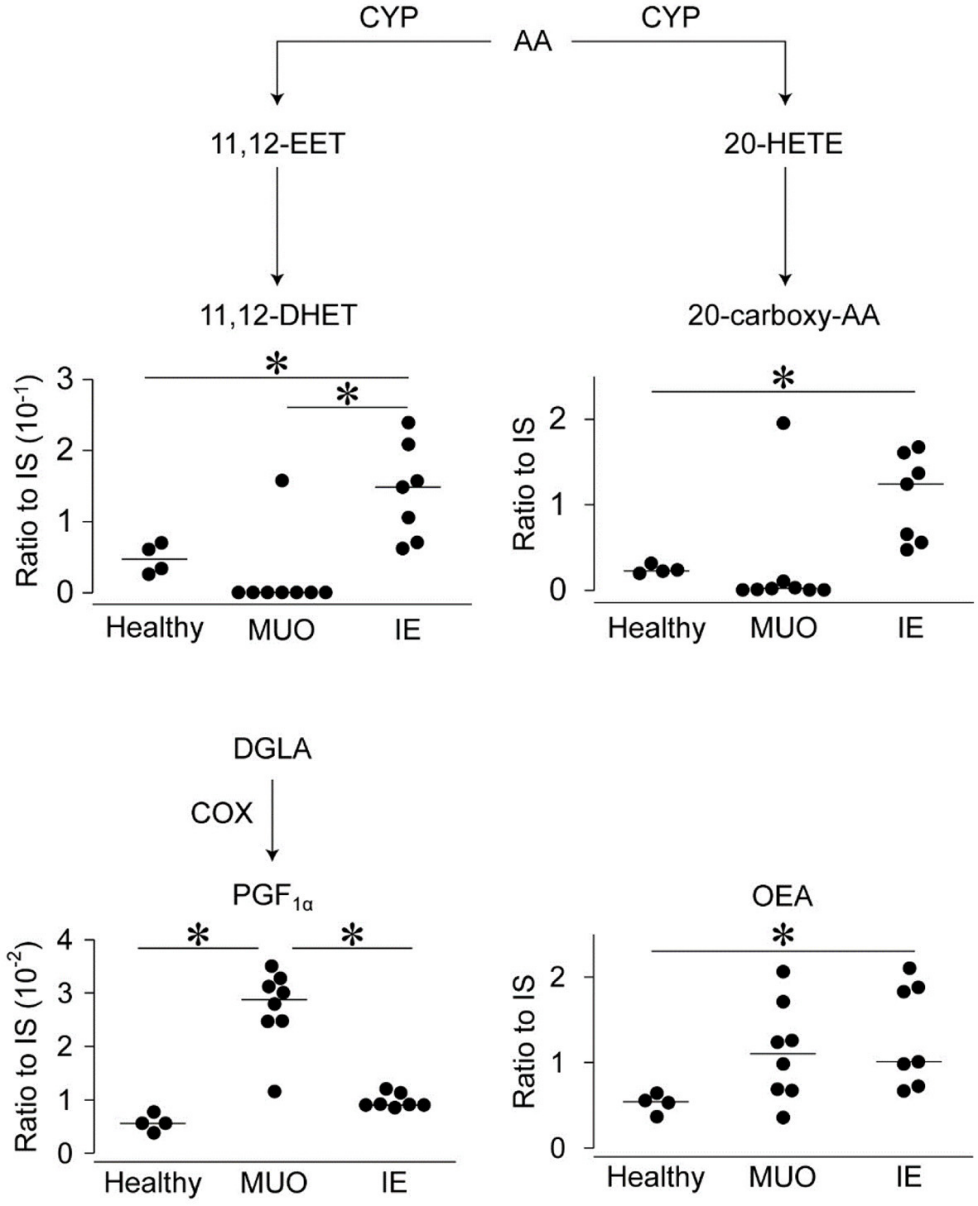
Levels of lipid metabolites in the plasma of dogs with central nervous system (CNS) diseases on the brief lipid metabolic map. The levels of lipid metabolites, which were significantly different between the groups, are shown on the brief map. Dots represent individual values, and horizontal bars represent the medians of each group. All values are represented as ratios to internal standards (IS). Abbreviations on the side of arrows indicate the mediators of those pathways. AA, arachidonic acid; COX, cyclooxygenase; CYP, cytochrome P450; DGLA, dihomo-gamma-linolenic acid; DHET, dihydroxyeicosatrienoic acid; EET, epoxy-5Z,8Z,14Z-eicosatrienoic acid; HETE, hydroxyeicosatetraenoic acid; IE, idiopathic epilepsy; OEA, oleoylethanolamide; MUO, meningoencephalomyelitis of unknown origin; and PGF1_α_, prostaglandin F1 alpha. *indicates statistical significance at *p* < 0.05.

Of the 114 lipid metabolites detected in plasma, the levels of only four were significantly different between the groups (Figure 2). The plasma levels of 20-carboxy-AA were higher in dogs with IE than in healthy controls but not in those with MUO. Similarly, the plasma levels of oleoylethanolamide (OEA) and 11,12-dihydroxyeicosatrienoic acid (11,12-DHET) were greater in dogs with IE than in healthy controls. Moreover, the levels of 11,12-DHET were higher in dogs with IE than in dogs with MUO. The plasma levels of PGF_1α_, a metabolite of dihomo-gamma-linolenic acid, were significantly higher in dogs with MUO than in those with IE and healthy controls (*p* < 0.05).

### 3.4 Production of NO in CSF or plasma

**Figure 3:**
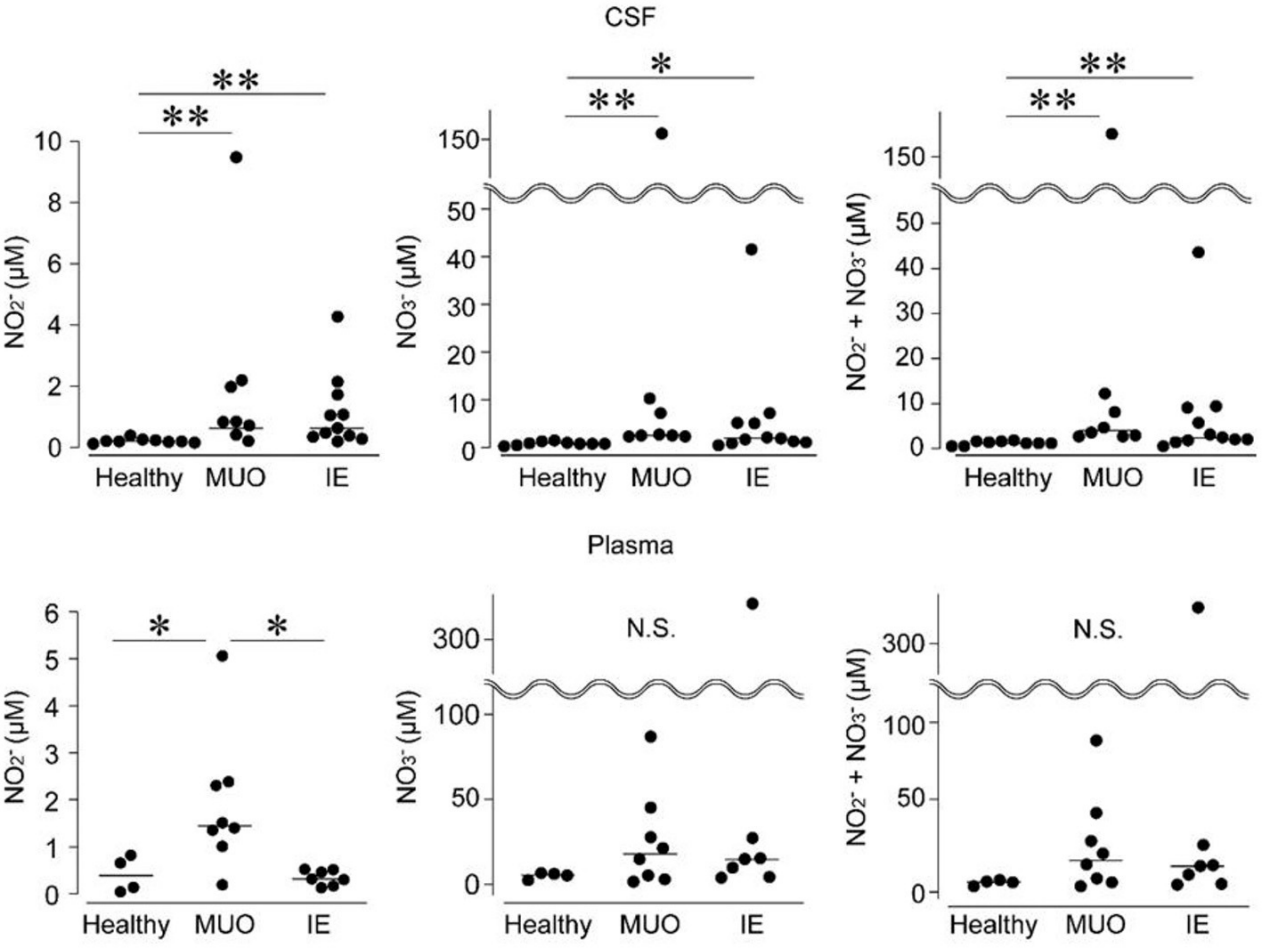
Levels of nitrogen oxide (NO) production in the cerebrospinal fluid (CSF) and plasma of dogs with diseases of the central nervous system (CNS). IE, idiopathic epilepsy; MUO, meningoencephalomyelitis of unknown origin; and N.S., not significant. * and ** indicate statistical significance at *p* < 0.05 and 0.01, respectively.

Levels of NO production were assessed in the CSF and plasma samples (Figure 3). The CSF levels of NO${\;}_{2}^{-}$ and NO${\;}_{3}^{-}$ ions and their sum were higher in dogs with MUO or IE than in healthy controls. In contrast, although the plasma levels of NO${\;}_{2}^{-}$ were higher in dogs with MUO than in those with IE and healthy controls, there were no significant differences in NO${\;}_{3}^{-}$ levels or the sum of NO${\;}_{2}^{-}$ and NO${\;}_{3}^{-}$ among the groups (Figure 3).

### 3.5 Assessment of confounding factors

To assess the confounding factors, the lipid metabolites and NO data in each group were divided based on gender, treatment with or without prednisolone or AED medication, the frequency of seizures (more or less than 10 times a month), the position of the brain lesions (lesions in the cerebral cortex or in other positions), and the time elapsed since the last seizure. Some items could not be analyzed since the sample size of the subgroup was too small (*n* = 1 or 2). No significant differences were observed in all the cases (*n* = 3 or 4) that could be analyzed. In addition, the comorbidities were also shown in Supplementary Table 1. Two cases were diagnosed with cholestasis, one was diagnosed with diabetes mellitus (DM), hydrocephalus, hypothyroidism, and myxomatous mitral valve disease (MMVD). Medications prescribed were ursodeoxycholic acid for the cases of cholestasis, Insulin glargine for the case of DM, Isosorbide for the case of hydrocephalus, Levothyroxine for the case of hypothyroidism, and Pimobendane for the case of MMVD. When the metabolites and NOs were compared between the cases with or without comorbidities in each group, there were no significant differences. However, it's important to note that the statistical power of all these data was low.

## 4 Discussion

In this study, we compared the levels of various lipid metabolites and NO production in the CSF and plasma of dogs with MUO or IE with those in healthy dogs. The levels of several lipids increased drastically and changed between groups, suggesting that a few inflammatory reactions occurred in the brains of dogs in both groups and that there were differences in the quality and extent of the inflammatory reactions between the MUO and IE groups. In addition, lipid metabolites that showed characteristic changes in each group could serve as new biomarkers, enabling the differential diagnosis and interpretation of the pathophysiological metabolism of these diseases. The variations in the levels of these lipid metabolites are discussed below.

Lyso-PAF, which showed increased levels in dogs with MUO but not in those with IE, was the main metabolite of PAF. PAF is produced in various cells, including macrophages, platelets, and the vascular endothelium, following chemical or immune stimulation and plays a critical role in the pathogenesis of vasospasm and inflammation (18). As the levels of lyso-PAF correlated positively with the cell number in CSF, it could be derived from activated macrophages and inflammatory reactions. Moreover, PAF is a lipid mediator of neuronal and microvascular dysfunction secondary to ischemia and traumatic brain (19, 20). In human patients with subarachnoid hemorrhage, the levels of PAF and PAF acetylhydrolases increase in the CSF (21), suggesting that these may participate in the development of vasospasm. Hence, our findings indicate that the levels of lyso-PAF in CSF could be associated with cerebrovascular damage and the activation of macrophages derived owing to the onset of MUO and could serve as a marker for diagnosing the diseases of CNS in dogs.

The levels of 6-keto-PGF_1α_, 20-carboxy-AA, and 9-HODE were also higher in the CSF of dogs with MUO than in healthy controls; however, their levels were not significantly different between dogs with MUO or IE. Few studies have focused on these metabolites in the brain diseases of any species. For example, 6-keto-PGF_1α_ is a major metabolite of PGI_2_ and is produced in high concentrations following the activation of vascular endothelial cells, and it is produced owing to endothelin-1 stimulation in human brain capillary endothelium cells (22). Moreover, 20-carboxy-AA and 9-HODE are lipoxygenase (LOX)- or cytochrome P450 (CYP)-mediated metabolites that may reflect inflammatory or redox reactions or both lipids may be involved in these diseases of CNS. However, we do not have reasonable evidence as to why only these specific lipids were detected among many LOX- and CYP-mediated metabolites. Future studies revealing new pathophysiological evidence are needed to explain these findings.

Oxidative stress is a biochemical state in which reactive nitrogen species are produced (23). The activation of NO synthase (NOS) and increased NO levels in the brain have been documented in rodent models of temporal lobe epilepsy (24). NOSs are expressed in neurons, endothelial cells, and immune cells, including microglia, and are termed nNOS, eNOS, and iNOS, respectively (12). The elevated NO levels in CSF in dogs with MUO or IE may be attributed to oxidative stress and NOS activation. Recurrent seizures have long-term effects on seizure susceptibility and the upregulation of nNOS expression in epileptic rat models (25). Moreover, accumulated lymphocytes and macrophages with an epithelioid morphology and the formation of granulomas around blood vessels are characteristic of brain lesions in MUO (26), implying that eNOS and iNOS can be activated in the MUO brain.

The plasma levels of several lipid metabolites are elevated in the diseases of CNS. The levels of 11,12-DHET in dogs with IE and those of PGF_1α_ in dogs with MUO were higher than those in healthy dogs and those afflicted by the other disease. Although the bioactivity of 11,12-DHET is not well documented, 11,12-EET, a substrate of 11,12-DHET, reportedly suppresses epileptic seizures by inhibiting glutamate release and opening a GIRK channel in CA1 pyramidal cells of the hippocampus (27). PGF_1α_ is a physiologically active lipid having various hormone-like effects and reportedly increases postcapillary resistance and capillary pressure in the canine lung (28). These findings suggest lipid metabolites may be linked to the pathophysiological mechanisms underlying the diseases of CNS. In addition, these alterations were observed in plasma, suggesting that PGF_1α_ could be an innovative biomarker for diagnosing CNS diseases without anesthesia or sedation, unlike that required for MRI or CSF collection.

Moreover, OEA, a fatty acid ethanolamine, is an endogenous peroxisome proliferator-activated receptor-alpha (PPAR-α) agonist (29), and it is a lipid mediator that alleviates obesity and hyperlipidemia (30). Recently, chronic intraperitoneal treatment with PPAR-α agonists reduced or abolished behavioral and electroencephalography expression of nicotine-induced seizures in mice and rats (31). Therefore, the activation of the PPAR-α pathway is being discussed as a new target of AED. Preclinical studies have shown that OEA is a potent anti-inflammatory and antioxidant with neuroprotective effects against alcohol abuse (32). In this study, OEA levels were high in the plasma of most dogs with CNS diseases. This finding suggests a biological defense mechanism where the levels of OEA could be upregulated in response to seizures, which plays a role in activating the PPAR-α pathway to alleviate the seizures.

This study has several limitations. All specimens were collected when dogs were diagnosed with MUO or IE. As a time-course examination was not performed, these data are only descriptive, and it is unclear whether these alterations could be the cause or effect of the diseases of CNS. All patients were diagnosed with MUO or IE based on clinical diagnostic criteria (8) and not on histopathological examination of their brain tissues with a definitive diagnosis. Age, body weight, and breed were statistically different between the groups, which could be due to selection bias. A few dogs had already been administered medicines, such as AED or glucocorticoids, and the number of elapsed days from the last seizure onset until sample collection varied. Owing to the small sample size of this study, we could not determine type II errors, assess the confounding factors or perform a stratified test or subgroup analysis substantially.

In conclusion, we identified several elevated lipid metabolites in the CSF and plasma of dogs with MUO or IE. A few lipid metabolites showed elevated levels in both dogs with MUO or IE. This study is the first report showing that lipid metabolites in CSF and plasma could be useful biomarkers for diagnosing the diseases of CNS and that lipid profile may be implicated in the pathophysiological mechanisms of these diseases. As canine spontaneous seizure diseases are suitable translational models for studying such diseases in humans, these results provide beneficial information for human medicine.

## Data availability statement

The original contributions presented in the study are included in the article/Supplementary material, further inquiries can be directed to the corresponding author.

## Ethics statement

The animal studies were approved by the Veterinary Ethical Review Committee of the University of Tokyo. The studies were conducted in accordance with the local legislation and institutional requirements. Written informed consent was obtained from the owners for the participation of their animals in this study.

## Author contributions

TY: Writing – original draft, Supervision, Methodology, Investigation, Funding acquisition, Formal analysis, Data curation. ST: Writing – original draft, Investigation, Formal analysis, Data curation. TMo: Writing – review & editing, Methodology, Investigation. MM: Writing – review & editing, Investigation, Data curation. NN: Writing – review & editing, Validation, Methodology, Investigation, Formal analysis, Data curation. KK: Writing – review & editing, Investigation, Formal analysis, Data curation. MY: Writing – review & editing, Validation, Investigation, Data curation. TMu: Writing – review & editing, Writing – original draft, Supervision, Resources, Project administration, Methodology, Funding acquisition, Conceptualization.
