# Supplementary material for: Lipid metabolites and nitric oxide production in the cerebrospinal fluid and plasma of dogs with meningoencephalitis of unknown origin and idiopathic epilepsy: a pilot study
Source: Front Vet Sci. 2024 Jun 25;11:1397868. doi: 10.3389/fvets.2024.1397868 (PMC11232469; doi:10.3389/fvets.2024.1397868)
Supplement: Supplementary file 1 [file Table_1.DOCX]

Supplementary Material

# Supplementary Figure and Table

## Supplementary Figure


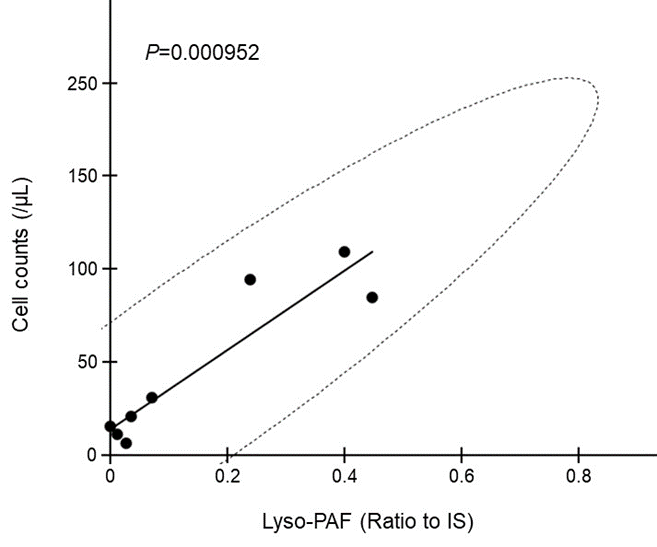


**Supplementary Figure 1.** Correlation between lyso-platelet-activating factor (lyso-PAF) levels and cell number in cerebrospinal fluid (CSF). Pearson's correlation analysis was performed using the XLSTAT software (version 2021.2.2.1141; AddinSoft, Paris, France). Lyso-PAF levels exhibited a positive correlation with cell number in the CSF of dogs with meningoencephalomyelitis of unknown origin (MUO) (*R*=0.926, *p*<0.001). The solid and dotted lines show the fitted regression line and variate normal ellipse, respectively (*p*=0.950).

Supplementary Table
